# Influence of Training–Testing Data Variation on ML-Based Deflection Prediction of GFRP-Reinforced High-Strength Concrete Beams

**DOI:** 10.3390/polym18010055

**Published:** 2025-12-24

**Authors:** Muhammet Karabulut

**Affiliations:** Department of Civil Engineering, Zonguldak Bulent Ecevit University, 67100 Zonguldak, Türkiye; karabulut@beun.edu.tr

**Keywords:** machine learning ML, three-point bending test, glass fiber reinforced polymer (GFRP) bar, reinforced concrete beam, flexural crack, K-Nearest Neighbors (KNN)

## Abstract

Glass Fiber Reinforced Polymer (GFRP)-reinforced concrete beams have gained significant prominence in structural engineering due to their advantageous mechanical and durability characteristics. However, the influence of training–testing data partitioning on machine learning (ML)-based deflection prediction for such members remains insufficiently explored. This study addresses this gap by evaluating the predictive performance of the K-Nearest Neighbors (KNN) regression algorithm in estimating the load–deflection behavior of GFRP-reinforced high-strength concrete beams. The experimental program comprised nine beams manufactured with concrete strength classes C45, C50, and C65, followed by ML-based deflection analyses using multiple data-splitting strategies. Findings indicate that the KNN model employing an 80:20 training–testing ratio provides the most accurate deflection predictions, achieving approximately 80% agreement with experimental results, while a higher prediction accuracy of approximately 85% was observed for beams with the highest concrete compressive strength (C65). Experimentally recorded deflections ranged from approximately 20 mm to values exceeding 50 mm, depending on the concrete strength class and loading level. Owing to its superior performance, the KNN model with an 80:20 training–testing ratio is recommended for predicting the deflection capacities of GFRP-reinforced high-strength concrete members. The study further examined the structural response associated with the use of GFRP as longitudinal tensile reinforcement. A consistent failure mechanism was observed across all beams, characterized by the formation of a single, wide vertical crack initiating at the beam’s soffit, regardless of concrete strength class. These observations contribute to a deeper understanding of the flexural behavior and fracture characteristics of GFRP-reinforced high-strength concrete beams and provide a foundation for future modeling efforts.

## 1. Introduction

Glass fiber–reinforced polymer (GFRP) bars have recently become a focal point of research as a potential substitute for traditional steel reinforcement in concrete members. This growing interest is largely driven by the inherent drawbacks of steel reinforcement, including its high density, pronounced susceptibility to corrosion in moisture- and chloride-rich environments, and the resulting long-term deterioration in mechanical capacity. In contrast, GFRP reinforcement offers several advantageous characteristics, most notably corrosion immunity, low self-weight, and high tensile strength—making it a promising alternative for applications where durability and reduced maintenance demands are critical.

In this study, GFRP bars were employed as longitudinal reinforcement, selected for their favorable balance between mechanical performance and cost efficiency. The flexural behavior of GFRP-reinforced concrete beams was evaluated through a comprehensive experimental program involving three-point bending tests on nine specimens. The beams were cast using concrete mixtures with varying compressive strengths, classified as high strength (C45, C50, C65), thereby enabling assessment of the influence of concrete strength on global flexural response.

With the rapid development of advanced computational technologies and artificial intelligence (AI), the integration of such tools into the construction domain is increasingly recognized as a means to enhance operational efficiency and shorten project delivery times. A further aspect that underscores the significance of the present research is the application of machine learning (ML) techniques to predict the flexural performance of GFRP bar–reinforced concrete beams using experimentally obtained data. In this context, the deflection response of the beams at their ultimate load-carrying capacity was evaluated through the application of K-Nearest Neighbors (KNN) regression models, enabling data-driven estimation of structural behavior under varying conditions. Moreover, investigating the deflection prediction accuracy of the KNN model across different training–testing data ratios constitutes another original contribution of this study.

Previous studies have experimentally investigated the deflection, cracking, and flexural behavior of GFRP-reinforced concrete beams under various loading and material conditions. Research on GFRP-reinforced fibered self-consolidating concrete beams under cyclic loading has shown that increasing polypropylene fiber content and reinforcement ratio improves serviceability by reducing crack propagation and deflections at both service and ultimate limit states [1]. The use of hybrid fibers and hybrid GFRP reinforcement systems in high-strength concrete beams has also been reported to enhance flexural performance, offering a balanced improvement in load-carrying capacity and deflection behavior while maintaining corrosion resistance [2]. In addition, experimental studies on beams incorporating recycled aggregate concrete, steel fibers, and GFRP reinforcement have demonstrated that the inclusion of steel fibers significantly increases first-crack load and improves overall flexural response compared with conventional reinforcement layouts [3]. Other investigations have focused on the load–deflection response, failure modes, and crack characteristics of GFRP-reinforced concrete beams, highlighting the influence of concrete strength and reinforcement ratio on flexural performance and crack control [4]. Experimental studies have shown that variations in concrete compressive strength and reinforcement ratio influence crack width and flexural performance, although failure is typically governed by concrete crushing and flexural failure, regardless of these parameters [5]. To enhance ductility, confinement of the compression zone using CFRP sheets and CFRP stirrups has been investigated, with findings indicating that CFRP stirrup confinement provides superior improvements in load-carrying capacity and ductility due to more effective concrete interaction [6]. The incorporation of fiber-reinforced concretes, such as PVA fibers, in conjunction with GFRP reinforcement has also been reported to significantly reduce crack development and enhance mid-span deflection, thereby improving overall flexural behavior [7]. In parallel, numerical and hybrid experimental–numerical studies on FRP–steel hybrid reinforcement systems and glass and basalt FRP bars have demonstrated that reinforcement configuration and ratio play a dominant role in governing stiffness, deflection, and failure mode, with FRP-reinforced beams often exhibiting concrete crushing rather than ductile behavior at higher reinforcement levels [8,9]. Furthermore, advanced modeling approaches incorporating bond interaction, prestressing effects, and partial composite action have been shown to accurately capture the full moment–deflection response of FRP-strengthened concrete beams when validated against extensive experimental datasets [10].

Experimental, theoretical, and machine learning (ML) regression analyses were conducted to evaluate the load–deflection capacities of reinforced concrete beams constructed with conventional steel reinforcement, innovative GFRP bar reinforcement, and varying concrete strength levels. The ML models demonstrated a high level of agreement with the experimental results, exhibiting accuracy rates of approximately 80–90% on average [11,12,13]. In addition, the flexural behavior of GFRP bar–reinforced members under elevated temperature conditions were also investigated experimentally [14].

Recent studies have increasingly employed machine learning (ML) and hybrid computational frameworks to predict the structural response and serviceability performance of reinforced concrete beams under diverse loading and degradation conditions. Advanced ML approaches, including interpretable AutoML, ensemble learning, and optimization-assisted algorithms, have demonstrated high predictive accuracy for key response parameters such as mid-span deflection, shear capacity, crack width, and failure mode, often outperforming conventional ML models and design-code formulations [15,16,17,18,19]. These studies consistently highlight the dominant influence of concrete strength, geometric properties, reinforcement configuration, and stiffness-related parameters on structural performance, while also emphasizing the value of interpretability tools (e.g., SHAP analysis) and practical predictive formulations. In parallel, experimental and numerical investigations on GFRP-reinforced concrete beams, including members constructed with normal- and high-strength concretes, have provided critical insight into flexural response and serviceability behavior, considering variations in reinforcement ratio, bar configuration, surface characteristics, and strengthening techniques [20]. Collectively, the literature underscores the growing potential of ML-based models for accurately capturing complex structural behaviors, while also revealing the need for focused studies that integrate high-strength concrete, GFRP reinforcement, and deflection-oriented ML prediction within a unified experimental–data-driven framework.

Extensive experimental and data-driven studies have investigated the flexural response, serviceability behavior, and deflection characteristics of reinforced concrete beams, including members reinforced with GFRP bars and subjected to diverse loading and environmental conditions. Experimental investigations on full-scale GFRP-reinforced beams have highlighted the significant influence of bar surface configuration, elastic modulus, and reinforcement ratio on bond behavior, cracking, and deflection response, while also revealing that commonly used design provisions—such as ACI 440.1R—tend to underestimate deflections at serviceability limit states [21,22]. These findings underscore the need for improved predictive models for FRP-reinforced concrete members.

In parallel, a growing body of research has employed machine learning (ML) and hybrid artificial intelligence frameworks to predict key performance indicators of reinforced concrete beams, including deflection, shear capacity, crack width, impact response, seismic performance, and long-term deformation [23,24,25,26,27]. These studies consistently demonstrate that ML-based models outperform traditional analytical formulations and design codes, achieving high predictive accuracy and strong generalization capability across a wide range of conditions. Critical parameters such as concrete strength, beam geometry, reinforcement configuration, loading characteristics, and degradation effects (e.g., corrosion and fire exposure) have been identified as dominant factors governing structural response. Furthermore, experimental and numerical investigations on strengthened and damaged RC beams, including members reinforced or retrofitted with CFRP systems, have provided valuable insight into the degradation mechanisms and post-strengthening performance under flexural loading [28]. The results demonstrate substantial reductions in flexural capacity and stiffness with increasing corrosion—showing only 52.5% residual strength at 25.6% corrosion—and a regression-based model is proposed to predict the flexural capacity of CFRP-strengthened, corrosion-damaged RC beams. The flexural behavior of reinforced concrete beams incorporating GFRP reinforcement and high- to ultra-high-strength concrete has been investigated through three-point and four-point bending tests, considering the effects of seawater exposure, abrasive surface conditions, and different types of concrete surface bond characteristics. The experimental results were analyzed with particular emphasis on the load–deflection response of the beams [29,30,31,32,33]. The flexural behavior of hybrid reinforced concrete beams combining FRP and steel reinforcement is examined through tests on twelve specimens, including steel-RC, GFRP-RC, and ten hybrid configurations, to assess the influence of FRP type, concrete strength, and effective reinforcement ratio [34]. The findings show that hybrid beams exhibit structural responses intermediate between steel-RC and GFRP-RC members, with flexural capacity enhanced by higher-modulus FRP bars or increased reinforcement ratio—albeit at the expense of ductility—while elevated concrete strength improves both capacity and ductility, and the proposed regression-based stress model demonstrates strong agreement with experimental results. The development of regression equations for estimating key performance factors of high-strength reinforced concrete beams with hybrid confinement—using cellular stirrups and externally bonded GFRP wraps—is presented based on experimental testing of six full-scale specimens [35]. Four-point bending results show that hybrid confinement enhances strength, deformability, and ductility, and the proposed regression models provide reliable predictions of the governing parameters. The flexural fatigue behavior of corrosion-damaged RC beams strengthened with an FRP-grid-reinforced ECC overlay is evaluated through cyclic testing, revealing that increased FRP grid content significantly extends fatigue life—while higher corrosion levels and load amplitudes reduce it—and that a regression-derived S–N model reliably predicts fatigue performance [36]. The flexural behavior of concrete beams reinforced with GFRP and CFRP bars is evaluated through four-point testing and code-based comparisons, revealing that ACI predictions align most closely with experiments and that variations in reinforcement ratio, bar type, and concrete strength govern deflection and cracking, while discrepancies in other standards arise primarily from neglecting tension stiffening and inaccurate cracking-moment formulations [37]. The deflection of reinforced concrete beams is modeled using an ICA-optimized ANN framework trained on 120 experimental datasets, and comparative analysis with five additional AI techniques shows that the proposed ICA-ANN model provides the most accurate and reliable predictions [38].

The flexural behavior of reinforced concrete beams with GFRP reinforcement in the tension zone, particularly when high-strength concrete is used, has not been sufficiently investigated. While previous studies have mainly focused on strength and serviceability, aspects such as load–deflection response, failure mode characteristics, and crack development under three-point bending remain limited.

At the same time, machine learning (ML) techniques have been increasingly applied to predict the structural response of reinforced concrete elements. However, most existing studies adopt a single, conventional training–testing split ratio (typically 80:20), without examining its influence on prediction performance. The effect of different data partition strategies on ML-based deflection prediction remains largely unexplored.

This study addresses these gaps by providing experimental insight into the flexural response of GFRP-reinforced high-strength concrete beams and by systematically evaluating the effect of different training–testing split ratios on ML-based deflection prediction. The results highlight the sensitivity of ML performance to data partitioning and contribute to a more transparent and reliable application of ML methods in structural engineering.

This study experimentally investigated the failure behavior and crack formation patterns of GFRP-reinforced concrete beams with identical reinforcement details under different high concrete compressive strength levels, and the deflection values of the RC beams were determined experimentally. To predict the deflection values of GFRP-reinforced concrete beams at ultimate load-carrying capacity, the K-Nearest Neighbors (KNN) machine learning method was employed. The training–testing data ratios of the KNN model that yielded the most accurate results were examined, and the optimal ratio was recommended for deflection prediction in GFRP-reinforced concrete beams.

## 2. Materials and Methods

### 2.1. Glass Fiber Reinforced Polymer (GFRP) Bar

**Figure 1:**
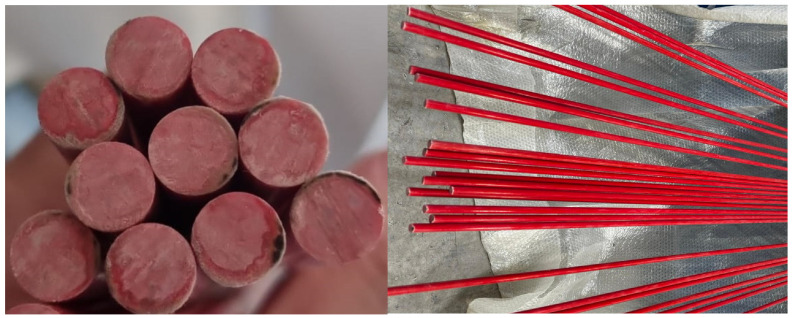
Cross-section and longitudinal GFRP bar reinforcement.

GFRP-reinforced concrete beams containing tensile-zone GFRP bars consist of three primary constituents: concrete, steel reinforcement, and GFRP composite bars. The GFRP bars are manufactured using glass fibers with a linear density of 4800 Tex and are embedded within a polyester resin matrix. The geometric characteristics of the ϕ10 mm GFRP bars, including their cross-sectional and longitudinal configuration, are illustrated in Figure 1.

The mechanical characteristics of the GFRP reinforcement were evaluated through a combined experimental program involving both direct tensile testing and standardized three-point bending tests. The tensile behavior was characterized by using displacement-controlled uniaxial tests performed in accordance with ASTM D7205, employing precision-engineered grip assemblies to prevent slippage and eliminate premature grip-induced failures. Each tensile specimen had a calibrated gauge length of 400 mm and a 140 mm clear span between fixtures, and axial load–elongation data were continuously recorded until rupture. Complementing the tensile assessment, the flexural response of the GFRP bars was examined through three-point bending tests conducted on 150 mm-long specimens with a constant 100 mm support span. The bending evaluations revealed a linear-elastic response up to peak flexural capacity, followed by an abrupt post-peak strength loss characteristic of the brittle fracture mechanisms typical of polymer-matrix composite bars. The full set of mechanical parameters obtained from both test configurations—including tensile strength, elastic modulus, rupture strain, and flexural capacity—is provided in Table 1.

**Figure 2:**
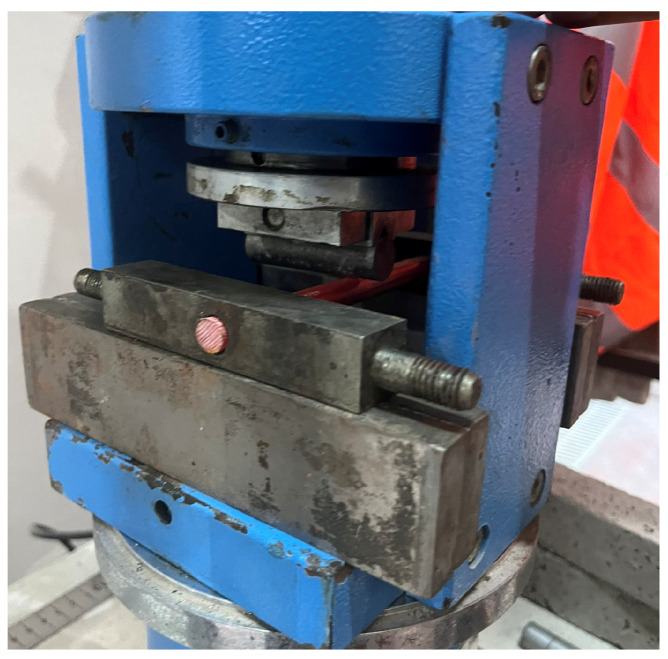
Three-point bending test setup of GFRP bar samples [12].

Figure 2 shows the 3-point bending test setup of GFRP bar samples. Tests were performed with a loading rate of 30.5 Hz.

**Figure 3:**
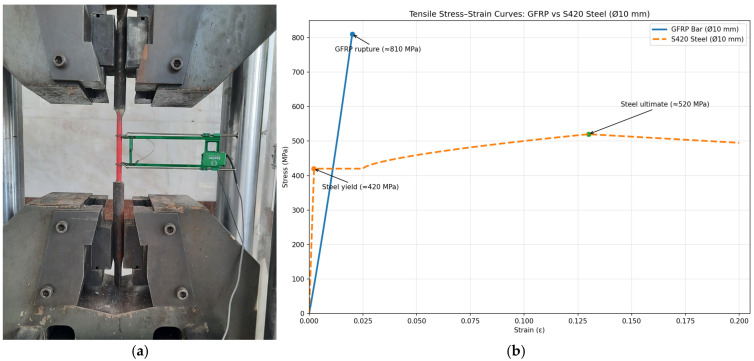
Tensile tests: (**a**) GFRP bar specimens and uniaxial tensile test setup; (**b**) test results.

In addition to the flexural tests conducted to determine the mechanical properties of the GFRP bar specimens, axial tensile strength tests were also performed, and the corresponding test setup is presented in Figure 3.

### 2.2. High Strength Concrete and Steel Bar

**Figure 4:**
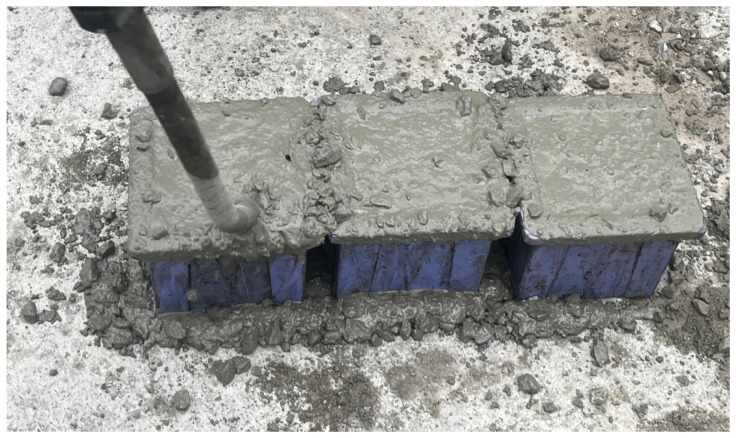
Cube sampling and vibration process for high-strength concretes.

In this research, three concrete categories—specifically the high-strength classes C45, C50, and C65—were employed. Cube specimens cast from fresh concrete during beam production and subsequently compacted through vibration are illustrated in Figure 4.

The concrete compressive strength results for the low (C45), moderate (C50), and high (C65) cube concrete sample groups, after 28 days of curing, are provided in Table 2. Cube specimens with dimensions of 150 × 150 × 150 mm were cast in standard molds.

The compressive strength corresponding to each concrete class—C45, C50, and C65—was quantified by averaging the test results of three companion specimens prepared and evaluated for each strength level. In this study, the compression zone of the beams was reinforced with two longitudinal steel bars of ϕ10 mm diameter, along with ϕ8 mm stirrups functioning as transverse reinforcement. Table 3 presents the mix proportions for the C45, C50, and C65 concrete classes.

### 2.3. Three-Point Bending Experiment of RC Beam

**Figure 5:**
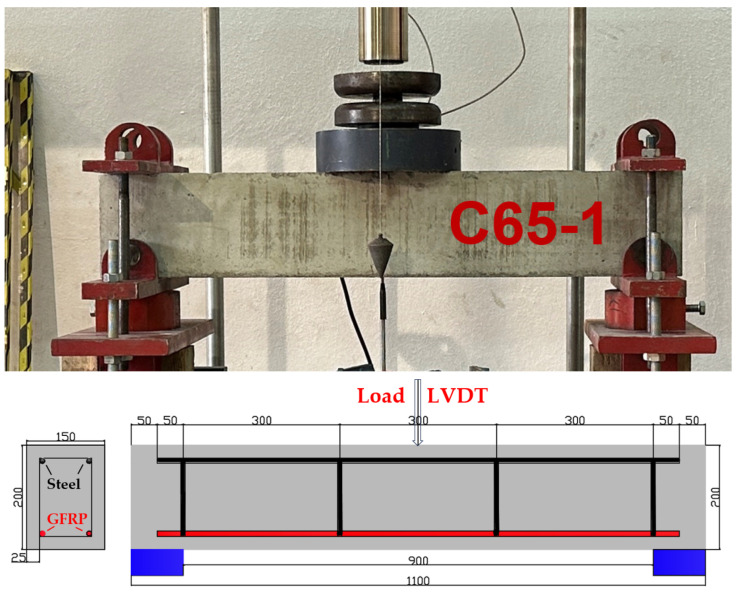
The configuration of the three-point bending test and the schematic representation of the beams incorporating GFRP longitudinal reinforcement in the tensile zone.

The configuration of the three-point bending experiment along with the schematic representation of the GFRP-reinforced beams is indicated in Figure 5. All nine GFRP bar–reinforced concrete beams produced for the study share identical dimensions, consisting of a width of 150 mm, a depth of 200 mm, and an overall length of 1100 mm. In each specimen, the longitudinal reinforcement placed within the compression zone comprises two steel bars, each 10 mm in diameter. The transverse reinforcement is provided by stirrups with a diameter of 8 mm, arranged at intervals of 300 mm along the beam length. In this study, two longitudinal GFRP bars with a diameter of 10 mm were used as reinforcement in the tension zone. A uniform concrete cover of 25 mm is maintained around the wrapped region on all sides of every reinforced concrete beam. The beams were subjected to three-point bending until structural failure occurred.

The principles of the three-point bending procedure, along with the mechanical relationships governing bending moment, flexural strength, stress distribution, and deflection in prismatic rectangular beams, are outlined in the following section. Flexural strength denotes the peak stress level reached by the member at the instant structural rupture occurs. The vertical deflection (Δ) is a function of both the material characteristics and the geometric attributes of the beam, including its cross-sectional form and clear span.

The load–deflection trace offers valuable information regarding the structural response, such as the degree of ductility, the extent of linear and nonlinear deformation regions, and the displacement associated with ultimate failure. Analytical expressions for the maximum bending stress (σ_max), maximum bending moment (M_max), maximum shear stress in a solid cross-section (τ_max), and the moment of inertia of a rectangular solid section (I_solid) are provided in Equations (1)–(4).

(1) $$\sigma_{max}=\frac{Mc}{I}=\frac{3FL}{2wh^{2}}$$

(2) $$M_{max}=\frac{FL}{4}$$

(3) $$\;\;\;\tau_{max}=\frac{3F}{2wh}$$

(4) $$\;\;\;\;\;I_{solid}=\frac{bh^{3}}{12}$$

### 2.4. Machine Learning-Based Deflection Prediction

**Figure 6:**
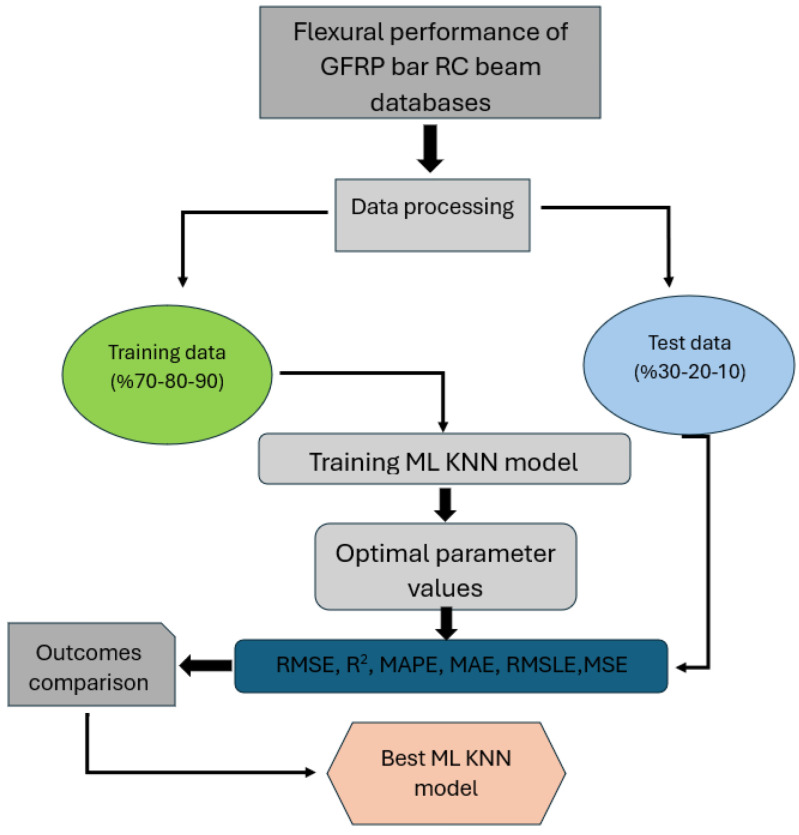
Structural overview of the ML-based analysis system and determination of the most effective KNN approach for predicting mid-span deflection.

Beyond the experimental program, a machine learning (ML) framework was utilized to characterize and forecast the mid-span deflection response of the reinforced concrete beams subjected to ultimate load levels. The architecture of the ML model implemented in this investigation is depicted in Figure 6.

The main objective of this research was to examine the capability of artificial intelligence techniques, particularly a K-Nearest Neighbors (KNN)-based machine learning regression model, to reproduce the nonlinear flexural response of reinforced concrete beams. Special emphasis was placed on understanding how the incorporation of GFRP bars as tensile reinforcement affects the bending performance of high-strength concrete beams. The dataset consisted of nine beams tested under three-point loading, and each data entry included variables such as concrete compressive strength (fc′), the type of longitudinal reinforcement (GFRP), geometric attributes of the beams (width, depth, span, and effective depth), reinforcement ratios, and the applied load. The predicted output parameter was the mid-span deflection (Δ) measured at the ultimate load stage.

**Figure 7:**
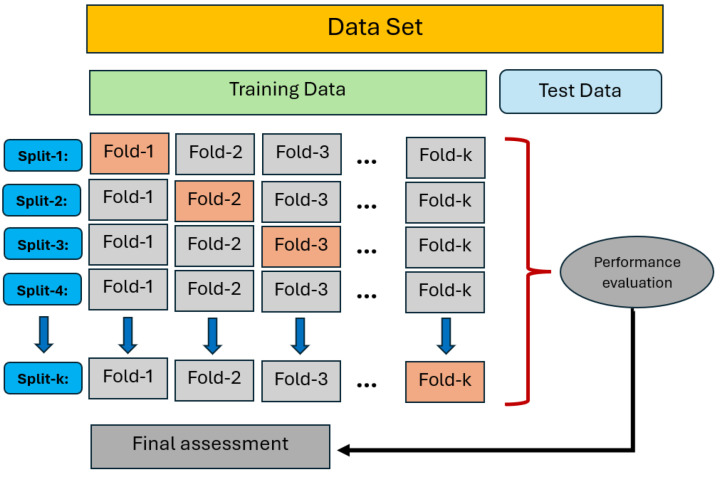
A cross-validation protocol consisting of ten iterative data partitions (K = 10) was implemented to rigorously assess the model’s predictive stability and generalization performance.

Machine learning analyses were conducted using the PyCaret environment to allow efficient model training and comparison. To determine which KNN regression configuration delivered the most accurate deflection estimates, the dataset was evaluated under three different train–test partitions: 70:30, 80:20, and 90:10. Additionally, 10-fold cross-validation was implemented to enhance model reliability and generalization capacity. The K-fold validation setup (K = 10) used in this study is illustrated in Figure 7.

The evaluation of the model’s predictive capability was carried out through several statistical indicators, including the Root Mean Square Error (RMSE), Mean Absolute Error (MAE), Mean Absolute Percentage Error (MAPE), and the coefficient of determination (R^2^). For a reliable regression model, reduced values of RMSE, MAE, and MAPE signify improved estimation accuracy, while an R^2^ value approaching 1.00 denotes a stronger correspondence between predicted and observed responses [13]. The mathematical expressions for these performance metrics are provided in Equations (5)–(8) [13].

(5) $$RMSE=\sqrt{\frac{\sum_{i=1}^{n}{\left(x_{i}-{x^{\prime }}_{i}\right)}^{2}}{N}}$$

(6) $$MAE=\frac{1}{N}\sum_{i=1}^{n}\left|x_{i}-{x^{\prime }}_{i}\right|\;$$

(7) $$R^{2}=1-\sum_{i=1}^{n}\;\frac{{\left(x_{i}-{x^{\prime }}_{i}\right)}^{2}}{\sum_{i=1}^{n}\;(x_{i}-{x^{\prime }}_{i})}$$

(8) $$MAPE=\sum_{i=1}^{n}\frac{1}{N}\left|{x^{\prime }}_{i}-\frac{x_{i}}{{x^{\prime }}_{i}}\right|\;$$

Only a limited number of studies have explored the application of PyCaret-based regression frameworks for estimating the strength, ultimate load capacity, and deflection behavior of composite materials; however, the available evidence demonstrates that such approaches can yield highly dependable results [39]. In this study, hyperparameter optimization was performed to enhance the predictive precision of the models, and both prediction-error diagrams and residual distribution plots were examined for selected beam specimens to provide a visual evaluation of model performance.

The incorporation of machine learning techniques offered significant advancements in interpreting structural response patterns and highlighted the capability of data-driven methodologies to serve as an effective supplementary tool to conventional experimental procedures in the analysis and design of structural systems.

## 3. Experimental and Machine Learning Analysis Results

### 3.1. Three-Point Bending Experiments and Results

The reinforced concrete beams were experimentally examined using a three-point bending configuration to assess their flexural response under static loading. Each specimen was simply supported over a clear span of 900 mm, and incremental loads were applied at the midspan through a hydraulic testing system. A high-precision displacement transducer positioned at the span center measured vertical deflections, while the applied load was continuously monitored by a calibrated load cell. The beams—measuring 150 mm in width, 200 mm in depth, and 1100 mm in total length—were carefully placed on the test frame to ensure accurate alignment with the supports. A spreader beam was utilized to provide uniform load transfer, thereby replicating realistic flexural loading conditions.

**Figure 8:**
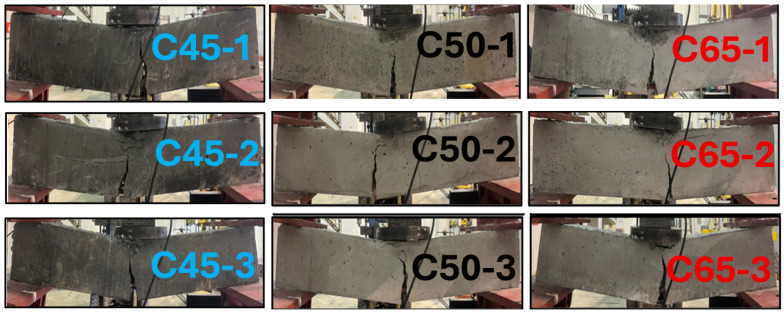
Three-point bending test of GFRP RC beams.

Testing began with the slow, controlled application of load, during which deflection data were captured in real time. Crack initiation, development, and propagation were visually monitored throughout the loading sequence, and the failure mechanisms were classified based on the observed cracking patterns. Each test concluded once the beam reached its ultimate load capacity and clear signs of structural failure were present. Key parameters—such as load–deflection behavior, peak load, maximum displacement, and failure characteristics—were documented for all specimens. Figure 8 shows the nine beams after testing and their post-failure appearance.

**Figure 9:**
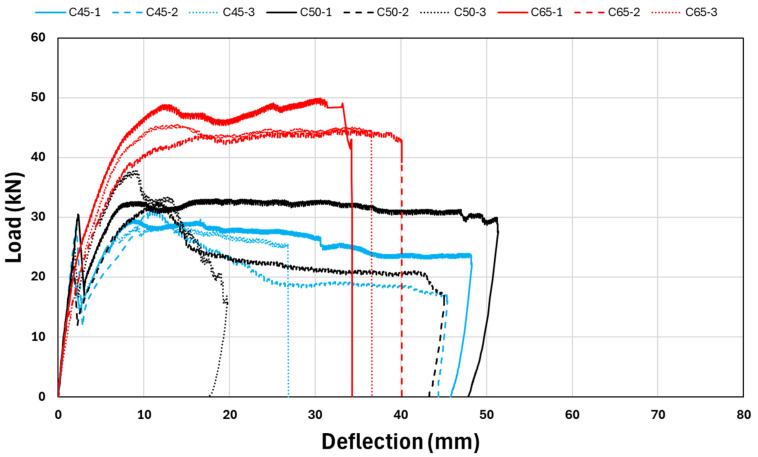
Three-point bending test results of RC beams.

Although the beams were identical in reinforcement layout and geometric properties, their compressive strength classes differed: low (C45), medium (C50), and high (C65). To allow a direct comparison of flexural performance across these strength levels, three beams were fabricated for each concrete grade, all reinforced with GFRP bars. This approach enables a clear evaluation of how concrete compressive strength influences the bending behavior of GFRP-reinforced high strength concrete RC members. Based on the experimental results, all beams exhibited a similar failure mechanism—crushing of the compression zone accompanied by the formation of a wide, dominant flexural crack originating from the midspan at the tension face—regardless of the concrete strength category. The three-point bending test results of 9 GFRP reinforced high concrete strength beams are given in Figure 9.

Based on the three-point bending test results, the C65 group of reinforced concrete beams—featuring the highest concrete strength and GFRP reinforcement—achieved the greatest load-carrying capacity. The average ultimate load of specimens C65-1, C65-2, and C65-3 was found to be approximately 46 kN. Furthermore, it is evident from Figure 9 that the highest ultimate load capacity, around 50 kN, was attained by specimen C65-1. In general, although the beam group with the highest concrete strength exhibited superior load-carrying capacity, its ductility level remained limited compared to the C45 and C50 beam groups.

Although the C45 and C50 beam groups, both reinforced with GFRP bars and characterized by higher concrete strengths, exhibited generally similar behavior under the three-point bending tests, the C50 group demonstrated a higher ultimate load-carrying capacity compared to the C45 series. Moreover, both the C45 and C50 groups showed a more ductile response overall when compared with the C65 beams, which possessed the highest concrete strength. Regardless of the concrete strength level, the crack formation pattern and mechanism in all beams were similar, initiating at the mid-span on the tension side and progressing as a wide, dominant flexural crack.

Despite having the lowest load-carrying capacity within the C50 group, specimen C50-1 exhibited the highest ductility among all tested beams. Following C50-1, the beam that demonstrated the second-highest level of ductility across the entire series was specimen C45-1.

Even though specimen C50-3 exhibited the highest load-carrying capacity within the C50 group, it demonstrated the most brittle failure mode among all beams. This behavior was interpreted as potentially arising from the heterogeneous nature of concrete.

Table 4 summarizes the detailed outcomes of the flexural testing conducted on nine concrete beams reinforced with GFRP bars. Interestingly, the C65 series—characterized by the highest concrete compressive strength—exhibited the lowest cracking load at the onset of flexural response among all GFRP-reinforced beam groups.

### 3.2. Machine Learning Systems Using K-Nearest Neighbors (KNN) Regression

The K-Nearest Neighbors (KNN) algorithm represents a widely employed classification procedure distinguished by its conceptual simplicity and methodological flexibility. Operating as a nonparametric technique, KNN determines the class of a query sample by identifying the most proximate instances within the feature space. As it does not presuppose any underlying probability distribution of the data, the method assigns labels to new observations based solely on their spatial proximity to established data points, exhibiting a conceptual alignment with certain clustering principles. Consequently, classification decisions are derived exclusively from the attributes of the input sample and the structure of the training set, without the need for an explicit parametric model or predefined functional form [40,41]. KNN falls within the category of instance-based, or “lazy,” learning techniques, as it does not construct an explicit model but instead retains all training observations and infers the class of a new sample through a voting procedure applied to its K most similar neighbors. The parameter K is crucial for determining predictive reliability; when K is chosen to be too small, the resulting estimates typically exhibit elevated variability [42].

The k-nearest neighbors (kNN) approach has long been applied in classification tasks owing to its practical effectiveness and straightforward interpretability [42]. Within this framework, an unlabeled instance is assigned a class based on the labels of its k closest reference samples in the feature space. As a lazy learning technique, kNN performs no explicit model construction; instead, it approximates the decision function locally and carries out computations only when a prediction is required. In the context of regression, the method estimates the response of a query point by taking the mean of the target values associated with its k nearest neighbors.

The similarity, or distance, between a test point $x_{t}$ and a training instance $x_{i}$ is commonly quantified using the Euclidean metric, expressed as follows (9) and (10):

(9) $$d\left(x_{t},x_{i}\right)=\sqrt{\sum_{n=1}^{N}{\left(x_{t,n}-x_{i,n}\right)}^{2}}$$

where N denotes the total number of features, and $x_{t,n}$ and $x_{i,n}$ represent the values of the n-th attribute for the test instance and the i-th training instance, respectively. The predicted response for $x_{t}$ is then obtained by computing the average of the target values corresponding to its k nearest neighboring samples:

(10) $$f\left(x_{t}\right)=\frac{1}{k}\sum_{i\in N_{k}\left(x_{t}\right)}\left(x_{i}\right)$$

where the set $N_{k}(x_{t})$ denotes the index collection corresponding to the k nearest neighboring samples of $x_{t}$.

The study investigates a broad spectrum of structural parameters—including various levels of concrete compressive strength, particularly within high-strength concrete classes—as well as GFRP reinforcement materials and changes in beam width, height, effective depth, and reinforcement ratios. In the context of statistical evaluation, a set of widely recognized indicators is utilized to quantify the predictive capability of machine learning models. Among these are the Root Mean Squared Logarithmic Error (RMSLE), Root Mean Square Error (RMSE), Mean Absolute Error (MAE), Mean Squared Error (MSE), the coefficient of determination (R^2^), and the Mean Absolute Percentage Error (MAPE). Together, these performance metrics provide an impartial assessment of how accurately the regression-based ML model’s estimations correspond to the actual measured values.

Although the prediction of composite strength, ultimate load-carrying capacity, and deflection behavior using PyCaret-based regression approaches has been explored only to a limited extent, existing studies report highly consistent analytical outcomes [39]. In the present work, MAE, MSE, R^2^, RMSE, and RMSLE metrics were computed through three distinct KNN regression procedures to predict the ultimate load–deflection responses of high-strength concrete beams reinforced with GFRP bars. Among these analyses, the KNN regression model that most accurately reproduced the experimental findings was selected as the reference model. Table 5 outlines the input and output variables associated with the key parameters employed in the machine learning regression framework.

In the present study, it should be emphasized that each of the parameters listed in Table 5 plays a variable role in the deflection prediction results and in terms of identifiability, although their relative importance and level of influence are not the same.

Certain geometric parameters, such as span length, width, and height, were treated as constant variables. Variations in these parameters are beyond the scope of the present study, and therefore they were intentionally kept constant. It is nevertheless acknowledged that changes in these geometric properties would have a significant impact on the structural response.

The primary focus of this research is on investigating the influence of polymer reinforcement used in the tension zone on the flexural behavior of beams with high-strength concrete levels. For this reason, attention was directed toward material- and reinforcement-related parameters rather than geometric variability. However, the inclusion of geometric dimensions in the input dataset is both natural and appropriate, as they are fundamental descriptors of the structural system.

The analysis was conducted following a K = 10 repeated evaluation strategy, whereby the procedure was iteratively repeated. Within each repetition, the data were partitioned according to predefined training–testing ratios (e.g., 80:20). Importantly, model performance was not evaluated based on individual load–deflection points randomly sampled from the entire dataset, but rather on predictions made for beam data not used during the corresponding training phase. This evaluation strategy was applied separately for each beam specimen.

By assigning beam datasets exclusively to either the training or testing process within each iteration, no overlapping data points were shared between the training and test sets, thereby effectively preventing data leakage. Repeating the evaluation procedure multiple times further enhanced the robustness of the results.

This approach preserves the independence assumption at the specimen level and avoids the overly optimistic performance estimates that may arise from point-wise random splitting of highly correlated load–deflection data. While it is acknowledged that per-beam reporting may limit direct comparability with point-wise evaluation protocols, this strategy is considered to provide a more physically meaningful and conservative assessment of model generalization in the context of structural response prediction.

In Table 6, the predictive capability of the ML-based KNN regression model was examined for estimating the deflection values obtained from the three-point bending tests of GFRP-reinforced and high-strength concrete RC beams, using three different training–testing ratios. These three KNN models were constructed with training–testing splits of 70:30, 80:20, and 90:10, respectively. Upon evaluating the KNN model outcomes, it was observed that the 80:20 KNN model achieved the highest $R^{2}$ value most frequently, with a total of five times, followed by the 90:10 KNN model with three times, and the 70:30 KNN model with one time.

For the highest-accuracy deflection prediction obtained in the study, an $R^{2}$ value of 0.8648 was achieved using the 90:10 KNN model, corresponding to the RC beam with the highest concrete strength, coded C65-2.

The lowest-accuracy deflection prediction was obtained for the beam designated C50-3, with an $R^{2}$ value of 0.7088; however, when group averages are considered, the lowest mean accuracy was found in the C45 group.

**Figure 10:**
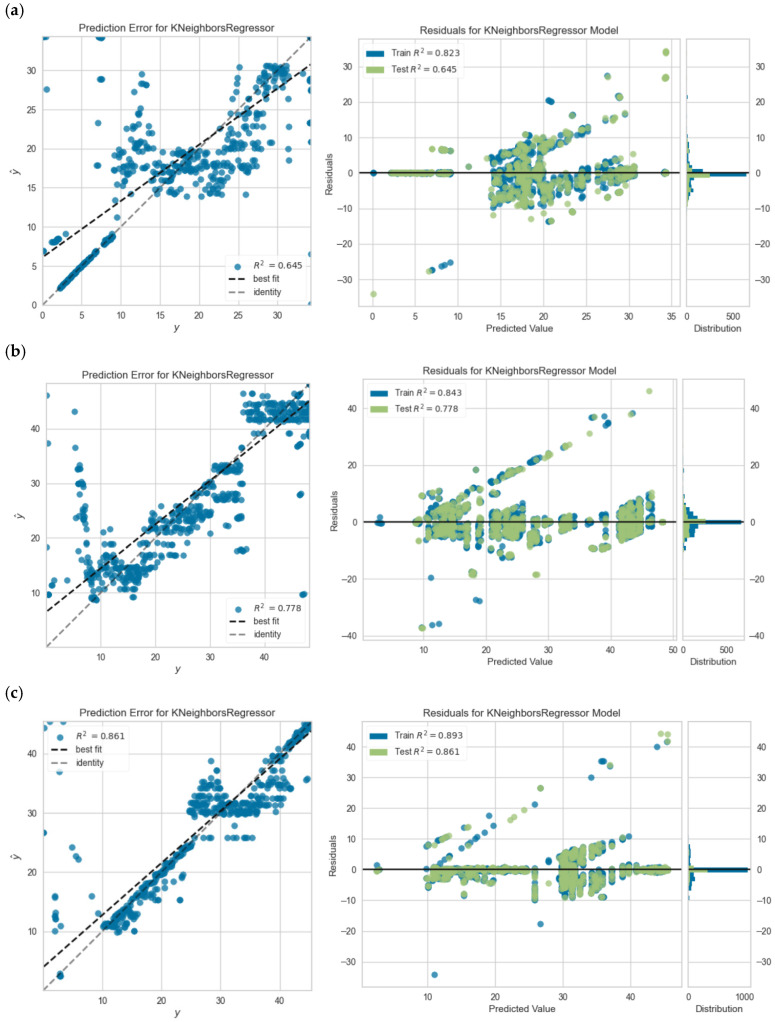
Prediction error and residual plots of (**a**) 70-30 KNN model for C45-1 beam, (**b**) 80-20 KNN model for C50-2 beam, (**c**) 90-10 KNN model for C65-2 beam.

Based on the analyses conducted, examples of prediction error plots and persistent prediction plots are presented in Figure 10 below.

**Figure 11:**
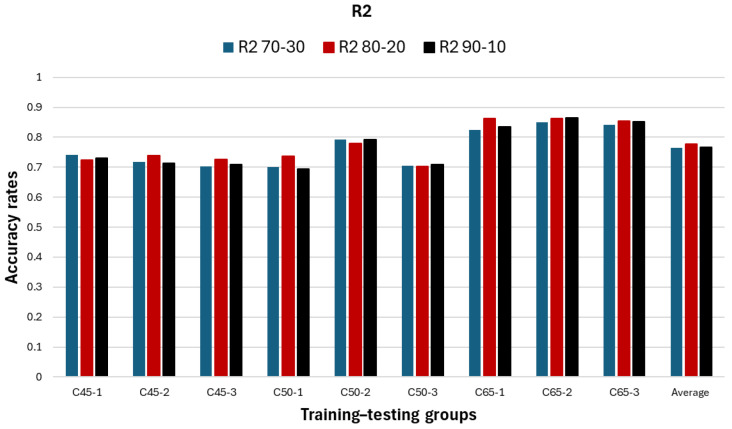
The results of the ML regression analysis performed for the deflection.

The comparative $R^{2}$ results of the 70:30, 80:20, and 90:10 KNN prediction models for the GFRP-reinforced and high-strength concrete beam groups are presented in Figure 11.

In comparison of the 70:30, 80:20, and 90:10 KNN models, it was observed that the prediction accuracies did not differ significantly among the three, and each model exhibited an average prediction accuracy of approximately 80%. Among them, the 80:20 model demonstrated slightly higher accuracy compared with the others. These findings indicate that, in the deflection prediction analyses of GFRP-reinforced and high-strength concrete beams, the ML-based KNN models provide comparable performance, with the 80:20 configuration offering a marginal advantage.

**Figure 12:**
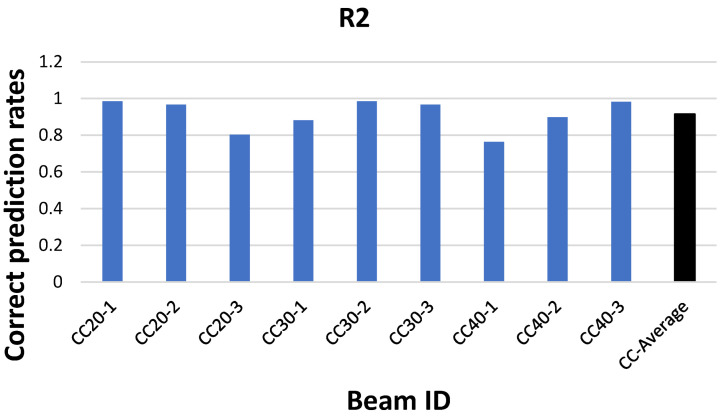
Results obtained from other studies [12].

The load–deflection behavior of reinforced concrete beams incorporating GFRP reinforcement in the tension zone and low-, medium-, and relatively high-strength concrete has been investigated, and the corresponding results are presented in Figure 12 [12].

The strong consistency between the results and those of the present study confirms the validity of the obtained findings.

Moreover, in a study employing a different machine learning approach to predict the load–deflection behavior of a total of 108 reinforced concrete beams incorporating GFRP, AFRP, CFRP, and BFRP reinforcement, with concrete compressive strengths ranging from 20 MPa to 117 MPa, both the experimental results and the machine learning predictions demonstrated a coefficient of determination (R^2^) of approximately 90%, indicating a high level of predictive accuracy [43].

## 4. Conclusions

In this study, the flexural behavior of GFRP-reinforced and high-strength concrete beams (C45, C50, and C65), as well as the predictive capability of KNN-based ML models with different training–testing ratios for estimating the deflection capacities of GFRP RC beams, were investigated through experimental tests and machine learning analyses. The key findings obtained from the study are as follows:

It was determined that the average predictive capacity of the KNN models was approximately 80% accuracy, and that the 80:20 model—commonly recommended in the literature—yielded the highest number of best deflection predictions. In particular, for GFRP-reinforced and high-strength concrete RC beams, the KNN model with an 80:20 ratio is recommended for deflection prediction.

The deflection predictions obtained from the ML-based KNN model for the RC beam group with higher concrete compressive strength (designated as C65) exhibited greater accuracy—approximately 85%—compared with the groups having relatively lower concrete strength.

Overall, although the C65 beam group—having the highest concrete strength—demonstrated a greater load-carrying capacity, its ductility was observed to be more limited compared to the C45 and C50 beam groups.

Among the C45, C50, and C65 beams, the minimum recorded deflection was approximately 20 mm, whereas the beam exhibiting the maximum deflection exceeded 50 mm.

The crack mechanism observed in the flexural behavior of high-strength reinforced concrete beams with GFRP reinforcement was found to be independent of the concrete compressive strength, consisting of a single, wide dominant crack forming at the mid-span region.

In high strength reinforced concrete beams where the tension zone is reinforced with longitudinal GFRP bars, the flexural crack development mechanism was observed to be largely unaffected by variations in concrete compressive strength. The failure pattern consistently manifested as a single, wide, and dominant flexural crack initiating at the mid-span region.

For concrete strengths of C100 and above, a separate study is required, and it is further recommended that other ML models be evaluated as well.

## Figures and Tables

**Table 1:** Mechanical properties of GFRP bars.

GFRP Bar Diameter (mm)	Cross Sectional Area (mm^2^)	Max. Tensile Strength (MPa)	Max Bending Load (kN)	Max. Bending Strength (MPa)	Ultimate Deflection Δ (mm)	Weight (gr/cm)	Support Distance (mm)
10	78.54	810	3.2	40.11	4.67	1.59	100

**Table 2:** Mechanical properties of concrete groups.

Concrete Group	7 Days Fc (Cube-MPa)	28 Days Fc (Cube-MPa)	28 Days Fc (Cylinder-MPa)
C45	37.1	55.8	44.8
C50	40.5	60.4	48.3
C65	55.8	82.6	66.2

**Table 3:** Mix proportions for the C45, C50, and C65.

Strength Class (MPa)	Cement (kg/m^3^)	Silica Fume (kg/m^3^)	Water (kg/m^3^)	Fine Agg. (0–4 mm) (kg/m^3^)	Coarse Agg. (4–16 mm) (kg/m^3^)	Superplasticizer %
C45	400	0	160	740	1060	1
C50	430	0	155	720	1050	1.2
C65	480	40	145	690	1030	2

**Table 4:** The outcomes of the flexural tests RC beams.

Groups	Beam Code	Initial Cracking Load, Fcr (kN)	Ultimate-Failure Load, Fexp (kN)	Maximum Mid Span Deflection, Δ exp (mm)	Ultimate Failure Moment, Mexp (kN·m)
C45	C45-1	22.8	29.5	48.1	13.28
	C45-2	28.8	33.1	45.6	14.90
	C45-3	22.4	28.4	28.2	12.69
C50	C50-1	30.3	33.4	52.1	15.03
	C50-2	22.6	32.3	45.4	14.54
	C50-3	25.7	37.6	19.8	16.92
C65	C65-1	43.1	50.1	34.8	22.55
	C65-2	39.4	44.5	36.6	20.03
	C65-3	40.6	46.4	40.3	20.88

**Table 5:** Statistical details of the parameters in the database.

Feature	Type	Cmin	Cmax	Ave
fc (MPa)	Input	44.8	66.2	53.1
fy (MPa)	Input	420	420	420
ff (MPa)	Input	794	819	810
b (mm)	Input	150	150	150
h (mm)	Input	200	200	200
d (mm)	Input	162	162	162
L (mm)	Input	1100	1100	1100
⍴fb (%)	Input	0.606	0.793	0.64
F (kN)	Input	29.5	50.1	39.8
Δ (mm)	Output	19.8	52.1	36

**Table 6:** Performance metrics of ML model.

Beam Name	Best Predictive ML KNN Model	MAE	MSE	RMSE	R^2^	RMSLE	MAPE
C45-1	70-30 KNN model	3.0045	31.9782	5.5871	0.7422	0.3895	1.9976
C45-2	80-20 KNN model	2.9800	32.6580	5.6655	0.7398	0.3706	1.6360
C45-3	80-20 KNN model	3.3073	33.5997	5.7373	0.7273	0.3867	1.8178
C50-1	80-20 KNN model	3.1944	49.1496	6.8911	0.7380	0.4655	1.7671
C50-2	90-10 KNN model	3.5235	43.5631	6.5675	0.7928	0.3951	0.5507
C50-3	90-10 KNN model	6.6062	98.6441	9.8824	0.7088	0.4410	0.5223
C65-1	80-20 KNN model	2.0539	21.1036	4.4769	0.8623	0.2914	0.9743
C65-2	90-10 KNN model	2.1324	20.7651	4.4685	0.8648	0.2793	1.1804
C65-3	80-20 KNN model	2.0648	22.2803	4.5857	0.8546	0.3178	1.0145

## Data Availability

The original contributions presented in this study are included in the article. Further inquiries can be directed to the author.
